# Artificial neural network applied to fragile X-associated tremor/ataxia syndrome stage diagnosis based on peripheral mitochondrial bioenergetics and brain imaging outcomes

**DOI:** 10.1038/s41598-022-25615-2

**Published:** 2022-12-10

**Authors:** Cecilia Giulivi, Jun Yi Wang, Randi J. Hagerman

**Affiliations:** 1 Department of Molecular Biosciences, School of Veterinary Medicine, University of California Davis, Davis, CA USA; 2 MIND Institute, University of California at Davis Medical Center, Sacramento, CA USA; 3 Center for Mind and Brain, University of California Davis, Davis, CA USA; 4 Department of Pediatrics, University of California at Davis Medical Center, Sacramento, CA USA

**Keywords:** Molecular neuroscience, Neurodegeneration, Cognitive ageing

## Abstract

No proven prognosis is available for the neurodegenerative disorder fragile X-associated tremor/ataxia syndrome (FXTAS). Artificial neural network analyses (ANN) were used to predict FXTAS progression using data from 127 adults (noncarriers and *FMR1* premutation carriers with and without FXTAS) with five outcomes from brain MRI imaging and 22 peripheral bioenergetic outcomes from two cell types. Diagnosis accuracy by ANN predictions ranged from 41.7 to 86.3% (depending on the algorithm used), and those misclassified usually presented a higher FXTAS stage. ANN prediction of FXTAS stages was based on a combination of two imaging findings (white matter hyperintensity and whole-brain volumes adjusted for intracranial volume) and four bioenergetic outcomes. Those at Stage 3 vs. 0–2 showed lower mitochondrial mass, higher oxidative stress, and an altered electron transfer consistent with mitochondrial unfolded protein response activation. Those at Stages 4–5 vs. 3 had higher oxidative stress and glycerol-3-phosphate-linked ATP production, suggesting that targeting mGPDH activity may prevent a worse prognosis. This was confirmed by the bioenergetic improvement of inhibiting mGPDH with metformin in affected fibroblasts. ANN supports the prospect of an unbiased molecular definition in diagnosing FXTAS stages while identifying potential targets for personalized medicine.

## Introduction

Carriers of fragile X messenger ribonucleoprotein 1 (*FMR1*) premutation alleles are those with 55–200 CGG repeats in the 5′UTR gene region, whereas those with > 200 CGG repeats have the full mutation. Premutation carriers may present with neurodevelopmental, emotional, and psychiatric problems, all with lower severity than those with the full mutation, which is associated with fragile X syndrome. Many adult male premutation carriers develop an age-dependent neurological disorder known as fragile X-associated tremor/ataxia syndrome (FXTAS)^1–3^. This disease is characterized by parkinsonism, intention tremor, cognitive-behavioral changes, and cerebellar ataxia, among other features. It may also present with lower limb proximal muscle weakness, peripheral neuropathy, and autonomic dysfunction^4^. Neurocognition may also be impacted, ranging from mild memory deficits to clear dementia, attention deficits, and impairments in declarative and procedural learning, executive functioning, and working memory^5–10^. Other symptoms may include anxiety, depression, irritability, reclusive behavior, and socially inappropriate behavior^7,8^.

Neuropathologically, FXTAS is an inclusion body disease because of the detection of eosinophilic ubiquitin-positive intranuclear inclusions in both neurons and astrocytes, especially in the hippocampus, frontal cortex, and cerebellar nuclei^11,12^. Consistent with the frontal cortex and hippocampal inclusions^11^, FXTAS involves frontal cognitive, learning, and recall deficits. Only confirmatory diagnoses of FXTAS are obtained by detecting inclusions in post-mortem brain samples.

In terms of progression, FXTAS, like other neurodegenerative diseases, proceeds in six successive stages of physical disability (tremor and balance problems, falls) and level of interference with activities of daily living^7^. The characteristics of each stage include the following: Stage 1—subtle or uncertain signs; Stage 2—minor, but clear, tremor and/or balance issues with minimal daily living activities’ interference; Stage 3—moderate issues with tremor and balance and at least occasional falls with significant interference of daily activities; Stage 4—severe tremor and balance problems requiring the use of a cane or walker aid; Stage 5—daily use of a wheelchair, and Stage 6—bedridden^7^. The diagnosis of FXTAS (definite, possible, probable) is established by neurological examinations and a combination of major and minor findings from clinical and MRI findings. Major FXTAS symptoms include (1) intention tremor of the hands, (2) gait ataxia, (3) detection of white matter hyperintensities (WMHs) by MRI in the middle cerebellar peduncles, and (4) FXTAS intranuclear inclusions in neuropathological studies. Minor FXTAS indications include (1) parkinsonism, (2) short-term memory and executive function deficits, (3) neuropathy, and (4) brain WMHs on T2-weighted MRI in the splenium of the corpus callosum, pons, and periventricular regions, along with generalized brain atrophy. Those with a definite FXTAS diagnosis are individuals with one major clinical symptom (1 or 2) and one major radiological finding (3 under major). Those with a probable FXTAS diagnosis have two major clinical signs or one minor clinical (from 1–3) and one major radiological finding (3). Those with a possible FXTAS diagnosis are individuals with one major clinical symptom and one minor radiological result^13^.

As indicated above, FXTAS diagnosis is based on neurological and MRI findings^14^. Some of these brain findings correlate with peripheral mitochondrial dysfunction^15^. The volume of white matter disease increases with FXTAS stages, following the trajectory or morbidity of the disease^15–17^; however, it has a low predictive value similar to the confirmatory diagnosis reached by the presence of brain inclusions.

Diagnosing FXTAS is challenging for clinicians because its phenotype overlaps with aging and other neurodegenerative diseases^18^. Furthermore, both onset and FXTAS disease progression are highly variable, and as of today, the progression of FXTAS trajectory cannot accurately be predicted. There is a great demand for early detection and more effective interventions to delay the onset of FXTAS or slow the course of its progression. Understanding the disease progression is a prerequisite for developing outcomes for predicting the course of FXTAS and determining effective intervention strategies.

Notably, no studies have used the power of combining molecular outcomes with clinical and imaging findings to develop a predictive model of FXTAS, even though this has been reported for Alzheimer’s disease^19^. At the molecular level and consistent with numerous reports on placing mitochondrial dysfunction at the center of initiation and propagation of cognitive and behavioral abnormalities in neurological/neurodegenerative diseases^20^, our research group was the first to report mitochondrial dysfunction (reflected as a shift from mitochondrial bioenergetics to glycolysis, a.k.a. the Warburg effect) in carriers of the premutation even before developing overt FXTAS symptoms^21–24^, results expanded by our team and others in a variety of biospecimens^21–23,25–29^ and mouse *Fmr1* premutation models^26,30,31^. Energy failure precedes the detection of the FXTAS hallmark, i.e., brain ubiquitin-positive intranuclear inclusions^11,12^, and some of the mitochondrial outcomes correlate with CGG repeats and severity of the phenotype^21,22,25^, highlighting the use of peripheral markers to mirror global mitochondrial dysfunction in disease-susceptible, post-mitotic tissues^32–37^. The brain is a highly aerobic organ, and within the cells that constitute the CNS, neurons rely heavily on mitochondrial ATP. Furthermore, considering the critical role mitochondria play in neurotransmitter metabolism^28,29,38^, it is not surprising that mitochondrial deficits likely compound the proteo- and RNA-triggered toxicities in CNS, contributing to FXTAS morbidity progression. This is well exemplified by our study of sulforaphane-treated fibroblasts from carriers at late stages whose bioenergetics were recovered by controlling their unfolded protein response and antioxidant capacities^39^. Although in vitro overexpression of the repeat-associated non-AUG (RAN)-mediated FMRpolyG product resulted in mitochondrial dysfunction^40^, no significant FMRpolyG levels in biospecimens from carriers have been found^41,42^ even with evident mitochondrial dysfunction.

Artificial Neural Network (ANN), a branch of artificial intelligence, allows the detection of pre-disease states based on early warning signals (for instance, mitochondrial dysfunction) prognosticating complex or multifactorial diseases of unexpected and large-scale critical passages in biosystems^43^. ANN’s detection of the “pre-disease state,” or in our case, progressing to more advanced FXTAS stages, is highly relevant because reversals are difficult or impossible to accomplish once the transition occurs, in contrast to relatively more direct intervention and prevention before the change occurs.

To this end, we turned to ANN to aid with diagnosing FXTAS stages. The ANN must first be “trained” by processing various input patterns and presenting each output result. The next step utilizes the trained ANN to recognize similarities under new input patterns and predict output patterns. In this way, ANN can detect early warning signs of crucial changes, defined as unexpected and large-scale state changes occurring in highly complex biological systems^43,44^. Based on our previous studies on early signs of mitochondrial dysfunction associated with executive dysfunction in carriers, we used ANN to extract similarities among a breadth of molecular and imaging outcomes and build a predictive model for FXTAS diagnosis and stage progression. In our model, disease development was considered as a critical change or transition^19,44–46^ even with noisy input data such as those originated from a diverse population of human participants (even if all are carriers) as opposed to those emerging from more homogenous populations (e.g., specific murine breed or strains; artificially controlled living conditions). The hypothesis of this study was to test whether the diagnosis of FXTAS stages can be aided by ANN analysis of mitochondrial outcomes, MRI findings, sex, and age and if this predicted diagnosis would have better predictive value than the use of more traditional diagnosis protocols.

## Results

### Study design and subject characteristics

**Figure 1:**
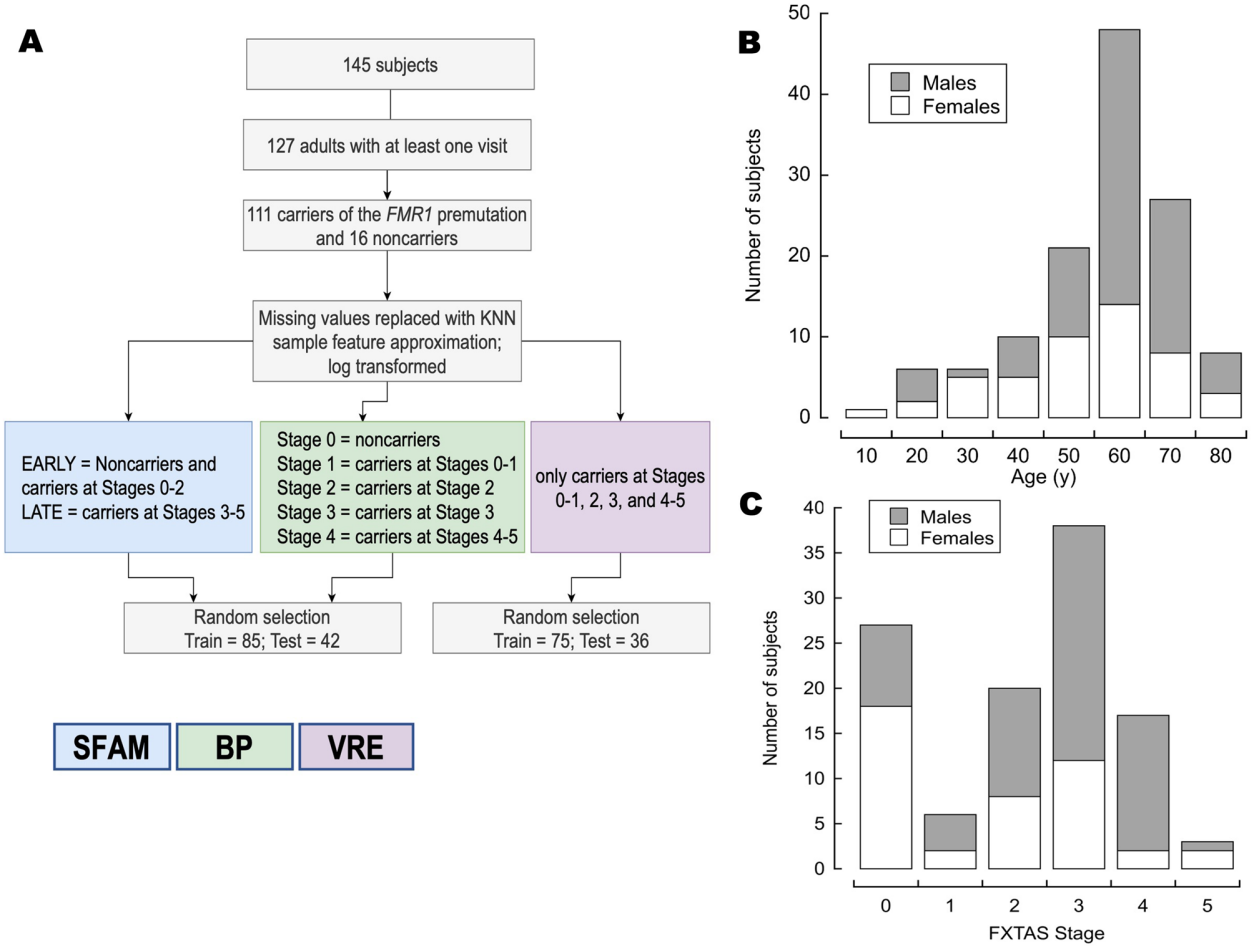
Experimental design and participants’ sex, age, and FXTAS stage distribution. (**A**) Scheme of the experimental design utilized in this study. From 145 eligible participants, we filtered those aged 18 years old and older with at least one visit to the MIND. The remaining 127 subjects were either carriers of the *FMR1* premutation (n = 111) or noncarriers (n = 16). Missing values were replaced by using the sample feature k-nearest neighbor algorithm, and the final values were log-transformed. The values were analyzed by using three different algorithms represented with colored boxes: blue, green, and purple boxes indicate that the data were tested with the simplified fuzzy adaptive resonance theory map (SFAM), backpropagation (BP), and visual rule extraction (VRE) algorithms, respectively. SFAM was used to predict EARLY vs. LATE FXTAS stages, a backpropagation algorithm to test numerical stage assignment, and VRE to unveil the combination of critical factors that predict stage assignment. More details were included in the text. Age and sex (**B**), and sex and FXTAS stage (**C**) distribution of the participants.

This study included 127 participants, 18 years and older, of both sexes, carriers (n = 111) and noncarriers (n = 16) of the *FMR1* premutation that had at least one visit to the MIND, with bioenergetic data from PBMC and fibroblasts, and MRI findings (Fig. 1). If more than one visit were available for a patient, only the first was used in the analysis. To maximize the statistical power, the small number of carriers at FXTAS Stage 1 (n = 6) were combined with those at Stage 0 (n = 27) and grouped under “Stage 1” (n = 33), whereas those at FXTAS Stage 5 (n = 3) were combined with those at Stage 4 (n = 17) and labeled as “Stage 4” (n = 20; Fig. 1A). The ratio of male to female carriers was 1.65 (79 males, 48 females) with a similar median age (median, range for males: 64, 18–86; for females 65, 25–86; Fig. 1B). As expected for an X-linked genetic disorder and consistent with other studies in the field^47,48^, women are usually less affected than men, with a median FXTAS stage lower than men (51% of male carriers at Stage 3 vs. 43% of females at Stage 0; Fisher exact test *p*-value (2-sided) = 0.004; Fig. 1C).

### ANN to diagnose FXTAS stages

The goal of this study was to utilize a neural network to discover patterns or a combination of imaging and bioenergetic outcomes that will allow categorizing carriers in FXTAS stages. A neural network is a software (or hardware) simulation of a biological brain (also named as Artificial Neural Network or "ANN"). The purpose of a neural network is to learn to recognize patterns in the data that are not apparent through a simple analysis. Consequently, software that learns through this process is named "Artificial Intelligence." The neural network must be "trained" by processing many input patterns resulting in outputs from each input pattern. Once trained, the neural network will make predictions by detecting similar patterns when presented with a new situation. The training of the ANN is a critical step in applying the ANN, as the predictions will rely on this training. To select a training set size, we performed a learning curve analysis^49^ by utilizing carrier status, FXTAS diagnosis, stage, sex, age, five MRI findings, and 22 units of mitochondrial outcomes for each cell type (PBMC and fibroblasts). Basically, a relatively small number of samples from our data was randomly chosen to train the ANN as detailed before^50^. The trained ANN was then used to predict the pattern of a randomly sampled test set not included in the training set. Then, iterative steps were taken to increase the size of our training sample while keeping that of the test set. By tracking the degree to which predictive accuracy on the fixed test set increased with the training set size, we obtained the needed size of training data until the differences between the network classifications and the clinical diagnoses became acceptable. The learning curve analysis was performed multiple times with different test sets to reduce variation in predictive accuracy. The resulting sample sizes were 85 subjects for training and 42 for testing.

The experimental outcomes included in our study were constituted by MRI imaging and bioenergetic findings. Of the five MRI imaging outcomes, two were binary variables constituted by the presence or absence of WMHs in the middle cerebellar peduncles and the cerebellum. The other three were the volumes of the WMHs, whole brain, and ventricles normalized by intracranial volume. These outcomes are supported by previous studies on brain MRI imaging changes associated with FXTAS progression, which include white matter lesions, generalized brain atrophy, ventricular enlargement, and accumulation of iron deposits in the subcortical nuclei^16,51–54^. While some of these changes can be detected before overt development of FXTAS symptoms, including loss of cerebellar and brain stem volume^55,56^, changes in white matter tracts^57^, and modifications in functional MRI recordings^58^, microstructural white matter disease in the middle cerebellar peduncles and the genu of the corpus callosum correlates with the severity of executive dysfunction and impairments in processing speed in carriers^59^.

The 22 bioenergetic outcomes included 11 from each cell type tested (PBMC and primary fibroblasts). Since many parameters can assess different aspects of mitochondrial dysfunction, here we provide a brief justification for the 11 chosen outcomes. Four of them were constituted by functional assays on ATP production sustained by various substrates and specific inhibitors allowing the evaluation of different segments of the electron transport chain. Among them, oxygen-linked ATP production was evaluated with glucose–glutamine in intact cells, and malate-glutamate, succinate, and glycerophosphate in permeabilized cells allowing mitochondrial access to these cell-impermeable substrates. Mitochondrial oxidative stress was estimated by evaluating the mitochondrial oxygen consumption under non-phosphorylating conditions (when ADP is limiting and its phosphorylation is almost null) normalized or not by the respiration with glucose–glutamine. Essentially these parameters reflect electrons leaking through the electron transport chain that, when combined with oxygen, generate reactive oxygen species (ROS)^60^. As inner membrane leakiness may impair ATP production, membrane integrity of mitochondria^61^ was assessed by evaluating the coupling between electron transfer and ATP production^62^. Under conditions of maximum oxygen uptake, the uncoupled respiratory control ratio (RCRu) is the ratio between maximal respiratory capacity, or State 3u, and the residual respiration after inhibition of ATP synthesis with oligomycin, or State 4o. In contrast, the ratio of phosphorylating to nonphosphorylating mitochondria is RCR. A critical parameter evaluated here was the spare respiratory capacity (SRC) This parameter is a key indicator of neuronal susceptibility to cellular stress^63^ and estimates the capacity of mitochondria to adapt to increasing energy demands. Even small decreases in spare or reserve respiratory capacity enhance neuronal vulnerability, predisposing the tissue to energy deficits and neurodegeneration^27^. This index was calculated as the ratio (in percentage) between the oxygen uptake under uncoupling conditions and that under glucose–glutamine. Finally, the activities of the terminal oxidase of the electron transport chain Complex IV and citrate synthase were evaluated. From all five Complexes, Complex IV is implicated in ataxia and other neurodegenerative diseases of all five complexes^64–66^. Citrate synthase is a Krebs’ cycle enzyme whose activity does not normally fluctuate with physiological or pathological conditions. As such, it can be used as a surrogate marker for mitochondrial mass^27,36,36,67^. Conversely, mtDNA copy number is an unlikely mitochondrial mass marker because it changes under several conditions and diseases resulting in either depletion (e.g., premature aging, failure to antagonize oxidative stress)^68^ or over-replication, as observed in FXTAS^23,27^ and others^23,27,69–77^.

To utilize ANN to analyze the data, we first applied a simplified fuzzy adaptive resonance theory map (SFAM) to predict a class, i.e., in our case, EARLY or LATE stages. SFAM uses numeric inputs (age, imaging and bioenergetic outcomes, sex, and carrier status as binary ones) to predict a class or category (i.e., EARLY or LATE). In lay terms, it provides a “fuzzy lookup” into the training database based on the similarity of coordinates within the multidimensional input space. For instance, if SFAM analyzes the parameters of a new FXTAS patient, it will return the diagnosis of the most similar pattern in the training data (early or late stage). For this algorithm, the ANN is constituted by a network of artificial neurons (also known as "nodes"). The strength of their connections is assigned a value (weight). Training begins with just one hidden node whose weights are set equal to the first record, and prediction is set equal to the class (e.g., Early) of the first record. Similarly, a new node is created whenever a new class is encountered (e.g., Late). The node whose weights best match the current input determines the Early or Late stage prediction if the degree of match exceeds the vigilance threshold value. If this prediction is correct, the weights of this winning node are adjusted according to this input. If the prediction is wrong or the vigilance threshold is not achieved, a new node is created with weights and predictions equal to this record. The algorithm learns through this iterative process justifying the label "Artificial Intelligence."

**Figure 2:**
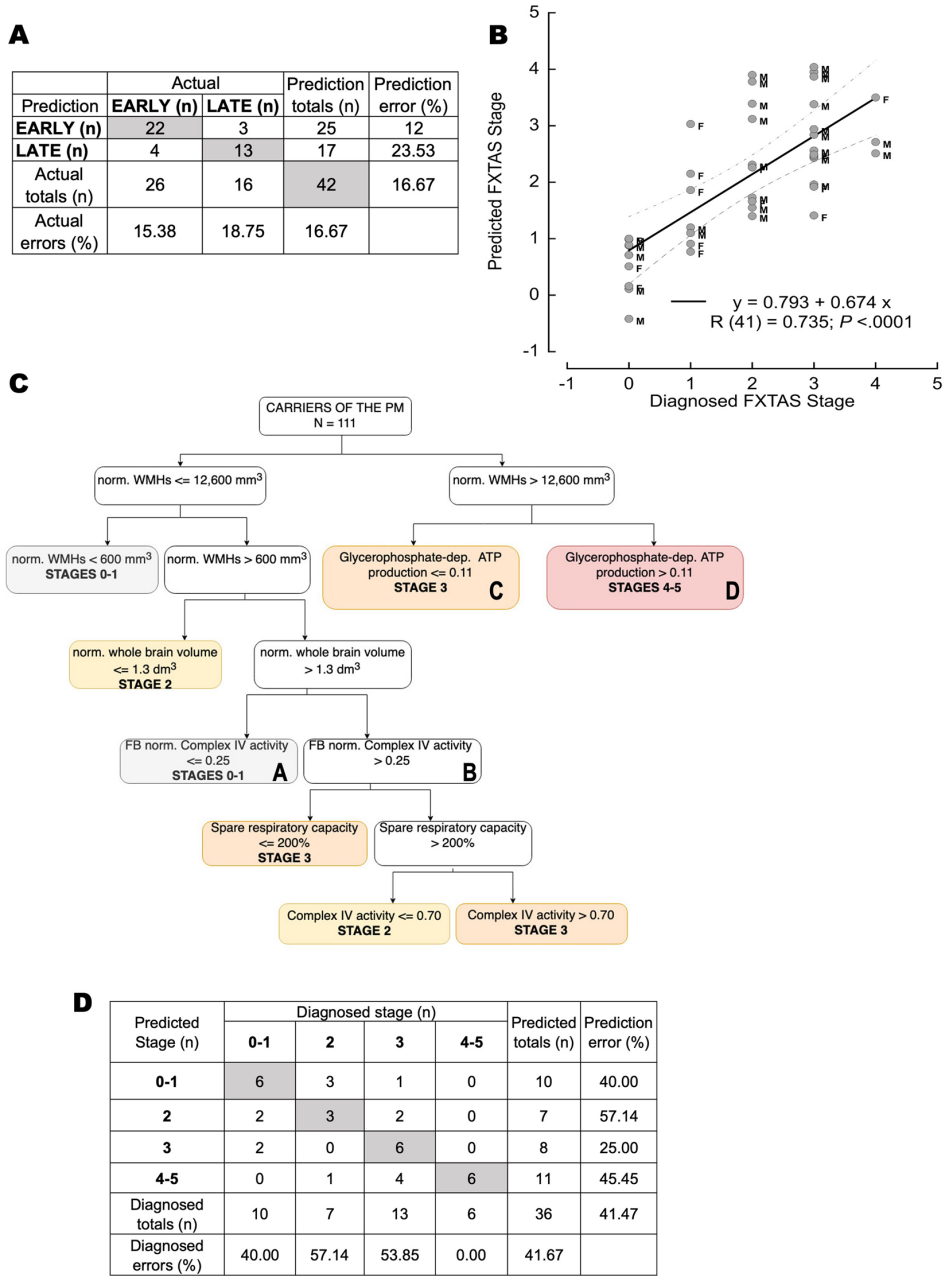
Artificial neural network prediction of FXTAS stages. The ANN utilized age, sex, imaging, and bioenergetic outcomes from PBMCs and fibroblasts from carriers and noncarriers to predict EARLY or LATE FXTAS stages by applying an SFAM algorithm (**A**), predicted FXTAS stage by utilizing the backprop algorithm (**B**), and VRE to identify key parameters that predict stage classification (**C**,**D**). Panel (**A**): Confusion matrix indicating the actual (diagnosed) stage and the predicted one (via SFAM). The number in the boxes are subjects in that category. In grey, subjects for which the diagnosed EARLY or LATE classification matched the predicted one. The leftmost column shows predicted errors, and the bottom row shows actual errors. Overall error is 16.67%. Panel (**B**): Data were analyzed by using the backprop algorithm. The results (diagnosed vs. predicted FXTAS stage) were visualized in a scatter plot, and a linear regression fitting was used. The correlation equation is shown; below it, the Pearson’s correlation is shown [R (degrees of freedom) = value; p-value]. The dotted line marks the 95% confidence interval for the regression; M and F, male and female, respectively. Panel (**C**): Decision tree obtained by analyzing the carrier data with the VRE algorithm. Abbreviations for imaging outcomes: *nv_WMH* normalized white matter hyperintensities and *nv_WB* normalized whole brain volume. For bioenergetic outcomes: GP-sustained ATP production = glycerol-3-phosphate-sustained oxygen-linked ATP production; CCO activity (or Complex IV activity) was expressed as nmol oxygen consumed × (min × million cells)^−1^, and the CCO/CS ratio = CCO activity normalized to that of citrate synthase used as a proxy for mitochondrial mass. SRC or spare respiratory capacity is calculated as the ratio of oxygen uptake under State 3u to that of State 3. It indicates the excess or reserve capacity of mitochondria to respond to stress (see more details under text). Those bioenergetic outcomes with the abbreviation FB were obtained with fibroblasts (e.g., FB CCO/CS), while all others refer to PBMCs. Colored boxes indicate those with stage prediction, Stages 0–1 in grey, Stage 2 in yellow, Stage 3 in orange, and Stages 4–5 in red. Boxes indicated with A, B, C, and D are used to discuss the differences in predicting the diagnosis of Stages 0–1 vs. 2–3 (A vs. B) and Stage 3 vs. 4–5 (C vs. D). Panel (**D**): Confusion matrix of diagnosed and predicted FXTAS stage resulting from VRE data analysis. Diagnosed and predicted stages ranged from 0–1, 2, 3, and 4–5. Box numbers indicate the number of subjects in that category. Boxes in grey indicate those matching the predicted and diagnosed stage. The leftmost column shows the prediction errors, whereas the bottom row shows the diagnosed errors. The overall error of the VRE analysis is 41.67%, with the most mismatches biased towards higher predicted stages.

In our dataset, *Early* stages included 9 noncarriers and 17 carriers at FXTAS Stages 0–2 (median age [95% CI] = 62.3 years [56–69]; 10 females and 16 males; 7 carriers at Stage 1 and 10 carriers at Stage 2) whereas *late* stages included 16 carriers at Stages ≥ 3 (median age [95% CI] = 64 [62–66]; 5 females and 11 males; 13 at Stage 3 and 3 at Stages 4–5; Fig. 1A blue box and Fig. 2A). This algorithm accurately predicted 84.6 and 81.3% of those at early and late stages, respectively. Overall, this classification resulted in an 83.3% sensitivity (95% CI 68.64–93.03), 100% specificity (95% CI 66.37–100), and an overall 86.27% accuracy (95% CI 73.74–94.30). The small number of mismatches between predicted and actual stages (n = 7, F/M = 2/5; 3 at late and four at early) resulted from the reclassification of 4 carriers diagnosed at early stages to late-predicted ones and the remaining three in the opposite direction.

Second, we analyzed the same test set of 42 participants (median age [95% CI] = 63 years [58–69]; 9 noncarriers, 7, 10, 13, and 3 at Stages 1, 2, 3, and 4; 15 females and 27 males) by using a backpropagation neural network (Fig. 1A, green box). This is the most common and robust type of neural network because solves classification problems. In our case, it assigned a numeric value to each diagnosed class (in our case stage), predicting a stage^78–80^, not a combined class (Early or Late) as it was when we used SFAM (vide supra). Training begins with the weights of all input parameter set to random numbers. For each input parameter, the predicted stage is compared to the actual stage, and the algorithm adjusts the weights to shift the predicted stage closer to the diagnosed one. Many cycles are made through the entire training data set, with the weights continually adjusted to produce more accurate stage predictions.

The analysis done by the backpropagation algorithm showed a strong and statistically significant association between the predicted and actual diagnoses of FXTAS stages (Fig. 2B). To calculate the error of this approach, we considered a mismatch between predicted and diagnosed stages if the absolute numerical difference was ≥ 2. Of the 42 participants, four carriers were identified as mismatches between the predicted and diagnosed stages (9.5% error). Most misclassified ones were the noncarriers (6 of 9 classified at Stage 1). The three carrier mismatches resulted from their reclassification to higher predicted stages (n = 3; M/F = 2/1). The sensitivity of this algorithm to detect the diagnosed stage was 87.88% (95% CI 71.80–96.60), with a specificity of 33.33% (95% CI 7.49–70.07), and an accuracy of 76.19% (95% CI 60.55–87.95).

Finally, a Visual Rule Extraction (VRE) algorithm^81,82^ was utilized to ascertain those factors (or their combination) important for diagnosing a stage that did not rely on clinical or neuropsychiatric evaluations. The algorithm behind this analysis (inductive rule extraction) is related to Machine Learning, Knowledge Discovery, Expert Systems, and Artificial Intelligence and is also called "Decision Tree Classification." This approach is based on the concept of entropy, a physicochemical term used to estimate randomness, disorder, or uncertainty in a population. Its use in artificial intelligence dates back more than 50 years ago^82,83^. Numeric and text input parameters (in our case, age, sex, stage, imaging and mitochondrial outcomes) are loaded to discover rules contained within the data to predict a class or category (in our case, stage). The VRE algorithm (based on an optimized version of the C4.5 algorithm^84^) builds the branches of the “decision tree” based on the lowest entropy (or randomness) or the most reliable information.

As with the previous ANN analyses, the VRE was trained with a stratified random sampling of 75 carriers of the *FMR1* premutation (14, 10, 38, and 13 at Stages 1, 2, 3, and 4; F/M = 28/47; median age [95% CI] = 66.2 years [63.5–68.9]), which represented roughly 1/3 of all carriers (Fig. 1A, purple box). The VRE algorithm analysis resulted in a decision tree with 15 nodes with an overall confidence of 41.67% (Fig. 2C,D) that relied on the following parameters: two outcomes from imaging, i.e., WMHs normalized by intracranial volume (nv_WMH) and normalized whole brain volume (nv_WB), and four bioenergetic outcomes, one from fibroblasts (Complex IV activity normalized to that of citrate synthase) and three from PBMCs (spare respiratory capacity, Complex IV activity, and glycerol-3-phosphate-sustained ATP production; Fig. 2C).

The decision tree indicated that WMHs normalized by intracranial volume are critical to distinguish carriers at early vs. late stages with those with a value ≤ 600 mm^3^ at Stages 0–1. Those with values between 600 and 12,600 mm^3^ may end up at Stage 2 if the normalized whole brain volume is ≤ 1.3 dm^3^. If higher and Complex IV activity normalized to that of citrate synthase in fibroblasts is ≤ 0.25, the carriers are classified in Stages 0–1. If this last ratio is higher than 0.25, the spare respiratory capacity in PBMCs up to 200% classifies carriers at Stage 3. If SRC > 200%, then the defining factor is Complex IV activity in PBMCs. If the lymphocytic activity of Complex IV is ≤ 0.70 nmol oxygen consumed × (min × 10^6^ cells)^−1^, carriers are at Stage 2, whereas if above this threshold, carriers are at Stage 3. Carriers with WMHs normalized by intracranial volume above 12,600 mm^3^ may end up at Stages 3 or 4–5 depending on the mitochondrial ATP production with the substrate glycerol-3-phosphate in PBMCs (> 0.11 nmol oxygen consumed ×  (min × 10^6^ cells)^−1^ at Stage 4 or ≤ 0.11 at Stage 3).

### Validation of ANN-mediated FXTAS stage diagnosis

We validated the above results from the decision tree using principal component analysis by utilizing all carriers (n = 111) and all outcomes (22 bioenergetic outcomes for each cell type, 5 MRI findings, sex, and age) vs. those six parameters selected by the VRE algorithm. To this end, we used principal component analysis because it allows analyzing large datasets with large numbers of parameters per subject by reducing the dataset complexity to increase the interpretability of data while preserving the maximum information. The data are linearly transformed into a new coordinate system where most of the data variation is observed with fewer dimensions than the original data (usually in a 2-dimensional plot) to visually identify clusters of closely related data points (in our case, stage clusters). The strategy was to use a statistical technique to visualize whether the VRE algorithm produced a better cluster separation of stages when using all vs. VRE selected parameters and utilizing all carriers, not just the test set.

**Figure 3:**
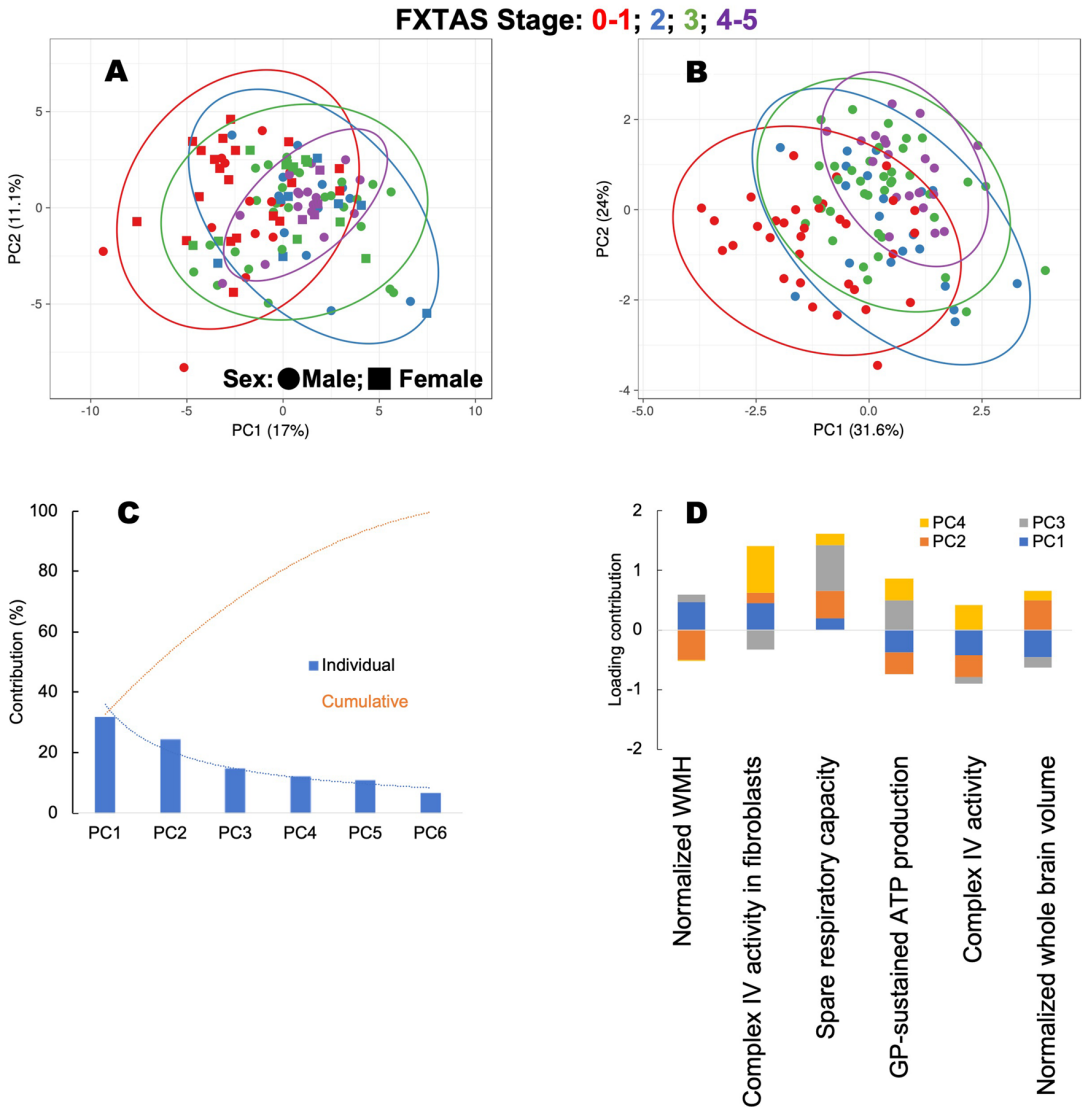
Principal component analysis of FXTAS stages based on sex, age, MRI findings, and bioenergetic outcomes. PCA (performed with ClustVis 2.0^119^) was applied to the test dataset by utilizing all 111 carriers and either all outcomes as input (panel **A**) or only those selected by VRE algorithm^81,82^ (panel **B**). Outcomes selected for panel B were taken from the decision tree in Fig. 2C. The data for constructing each PCA plot were scaled using a unit variance scaling applied to rows, and a singular value decomposition with imputation was used to calculate the principal component analysis method (for both panels). Other options were set as follows: no data transformation was used as the data were in the log form, no collapse of columns with similar annotations was performed, and no removal of constant columns was applied. (**A**) PCA of all subjects classified by stages by considering all parameters and outcomes (sex, age, diagnosed stage, and all bioenergetic and imaging outcomes). The X-axis and Y-axis show principal component 1 and principal component 2, which explain 17% and 11.1% of the total variance, respectively. The prediction ellipses demarcate the 0.99 probability that a new observation from the same group will fall inside the ellipse. (**B**) The sample analysis indicated under A was then performed only with the six (two imaging and four bioenergetic ones) outcomes selected by the VRE algorithm (from Fig. 2C). Using these parameters allows more precise separation of stages, as indicated by the larger variance contributions (principal component 1 and principal component 2 contributed to 31.6% and 24% of the total variance, respectively). Panels (**A**,**B**): Circles and square data points represent males and females, respectively; colors represented FXTAS stages: Stages 0–1 (red), blue (Stage 2), Stage 3 (green), and Stages 4–5 (purple). (**C**) A scree plot shows the individual contribution of the principal components (PC, blue bars) for explaining the variance in the data whose cumulative contribution (shown as a dotted orange line) accounts for 100% of the variance. PC1 and PC2 are the major contributors accounting for 57% of the variance. (**D**) A stack column plot shows the individual contribution of the six outcomes selected by the VRE algorithm on principal components 1–4 accounts for > 80% of the total variance. Imaging outcomes contribute mainly to PC1 (blue) and PC2 (orange); similarly, bioenergetic ones contribute to PC1-PC2 but also PC3 (grey) and PC4 (yellow). Bioenergetic outcomes were obtained with PBMCs except one indicated with “fibroblasts.” *PC* principal component.

When all carriers and parameters were utilized, no clear separation of the groups was noted in a principal component analysis score plot for the first two principal components. As indicated above, this plot is an orthogonal linear transformation that visualizes data to a new coordinate system. The greatest variance of the data lies on the first coordinate (X-axis) or first principal component (17%), with the second greatest variance (second principal component; 11.1%) on the second coordinate (Y-axis; Fig. 3A). Contrary to the analysis performed with all carriers and all parameters, when the six parameters identified by the VRE algorithm were used, better separation of the groups was achieved with larger variances in principal components 1 (31.6%) and 2 (24%; Fig. 3B). A scree plot was used to determine which parameters from those six VRE-selected ones needed to be retained as principal components to obtain the best separation of stages. This analysis indicated that all six were needed because considering the first two principal components, the cumulative variance contribution was close to 60% (Fig. 3C). Imaging and bioenergetic outcomes contributed mostly to principal components 1 and 2, but the bioenergetic ones also contributed to principal components 3 and 4 (Fig. 3D). These results indicated that the six parameters identified by the VRE algorithm were adequate to identify clusters of carriers at different stages even when using the larger set of carriers (n = 111).

### Biological implications of the VRE predictive value

**Figure 4:**
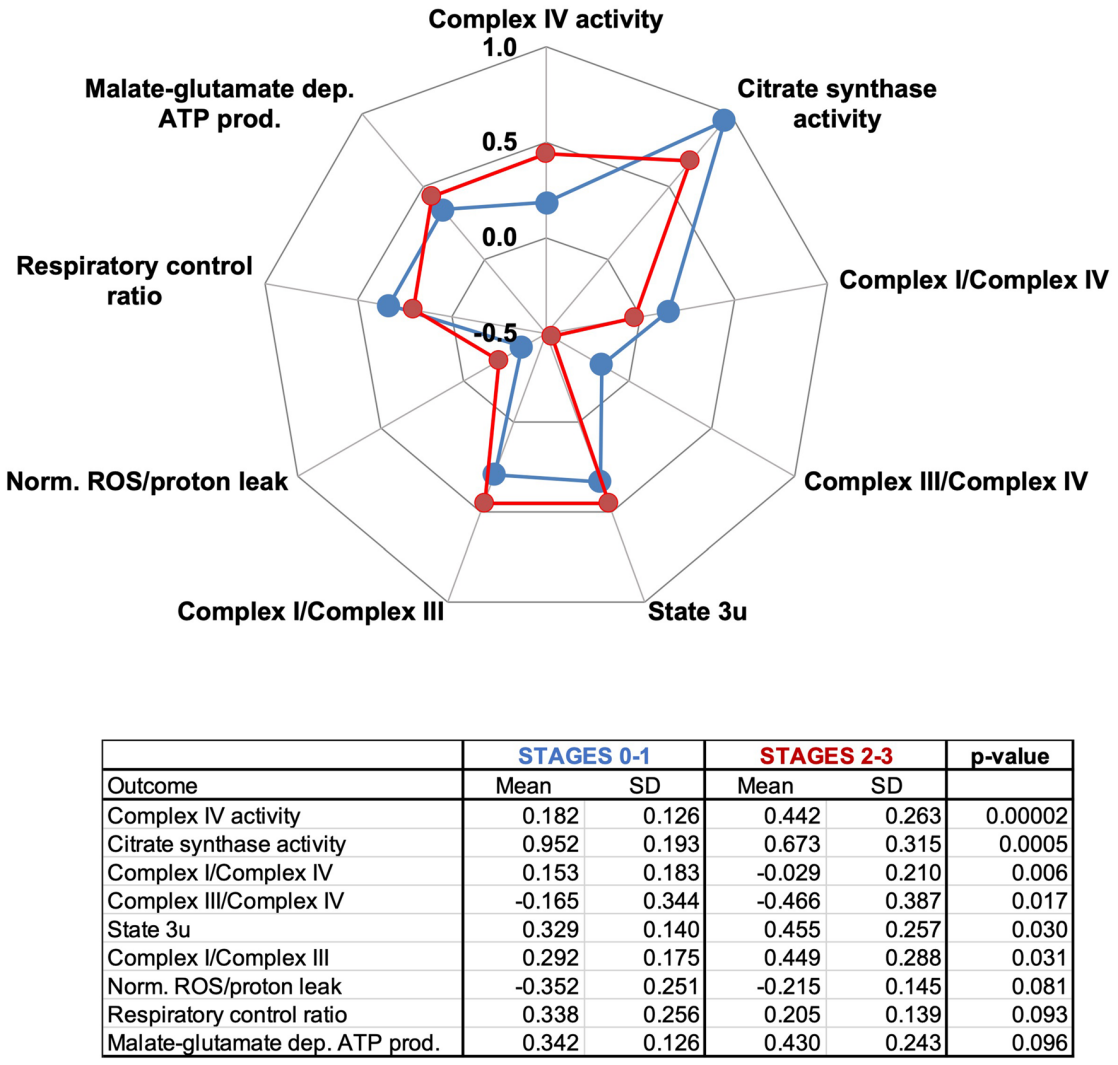
Bioenergetics of fibroblasts from patients at Stages 0–1 vs. 2–3. Top panel: Bioenergetic outcomes from primary fibroblasts obtained from carriers and assigned by the VRE algorithm as predicted Stages 0–1 (box A in Fig. 2C) or 2–3 (box B in Fig. 2C). The outcomes were plotted using a radar chart or spider web chart. For simplicity, only those with *p* ≤ 0.1 were shown—bottom panel: Statistical analyses performed using an unpaired two-tailed Student’s *t* test with unequal variances. Values were expressed as LOG FC.

Notably, the decision tree classification predicted that Complex IV activity in fibroblasts—normalized to that of citrate synthase—from participants at Stages 0–1 (Fig. 2C indicated as box A) was lower than in those at Stages 2–3 (Fig. 2C marked as box B). However, these results should not be interpreted as an “increase in mitochondrial function” at higher stages. A comprehensive analysis of mitochondrial bioenergetic data revealed that although Complex IV activity was higher in those at Stages 2–3 vs. Stages 0–1, citrate synthase activity was lower (Fig. 4). More importantly, malate-glutamate-fueled ATP production and the ROS and proton leak were higher in those at higher stages. Consequently, those at higher stages presented a lower coupling (RCR) and higher I/III and lower (I/IV and III/IV) Complex ratios (Fig. 4). The lower citrate synthase activity in fibroblasts from subjects at Stages 2–3 (by 30%) suggests lower mitochondrial mass. The other possibility (shrinking of Krebs’ cycle without changing the mitochondrial mass) does not seem viable as no changes in another Krebs’ cycle enzyme, succinate dehydrogenase or Complex II, were noted (Fig. 4 bottom panel; oxygen-linked ATP production sustained by succinate; *p* = 0.251).

This indicates that fibroblasts from carriers at Stages 2–3 vs. those at Stages 0–1 show lower mitochondrial mass, increased mitochondrial ROS, and an altered electron transfer across Complexes. This last observation is relevant because an altered mitochondrial and nuclear-encoded proteome (mitochondria-nuclear balance) reflects not only an inefficient ATP production because OXPHOS Complexes are not synthesized proportionally to each other, but also a protein imbalance that could lead to activation of the mitochondrial unfolded protein response (UPRmt)^85,86^.

**Figure 5:**
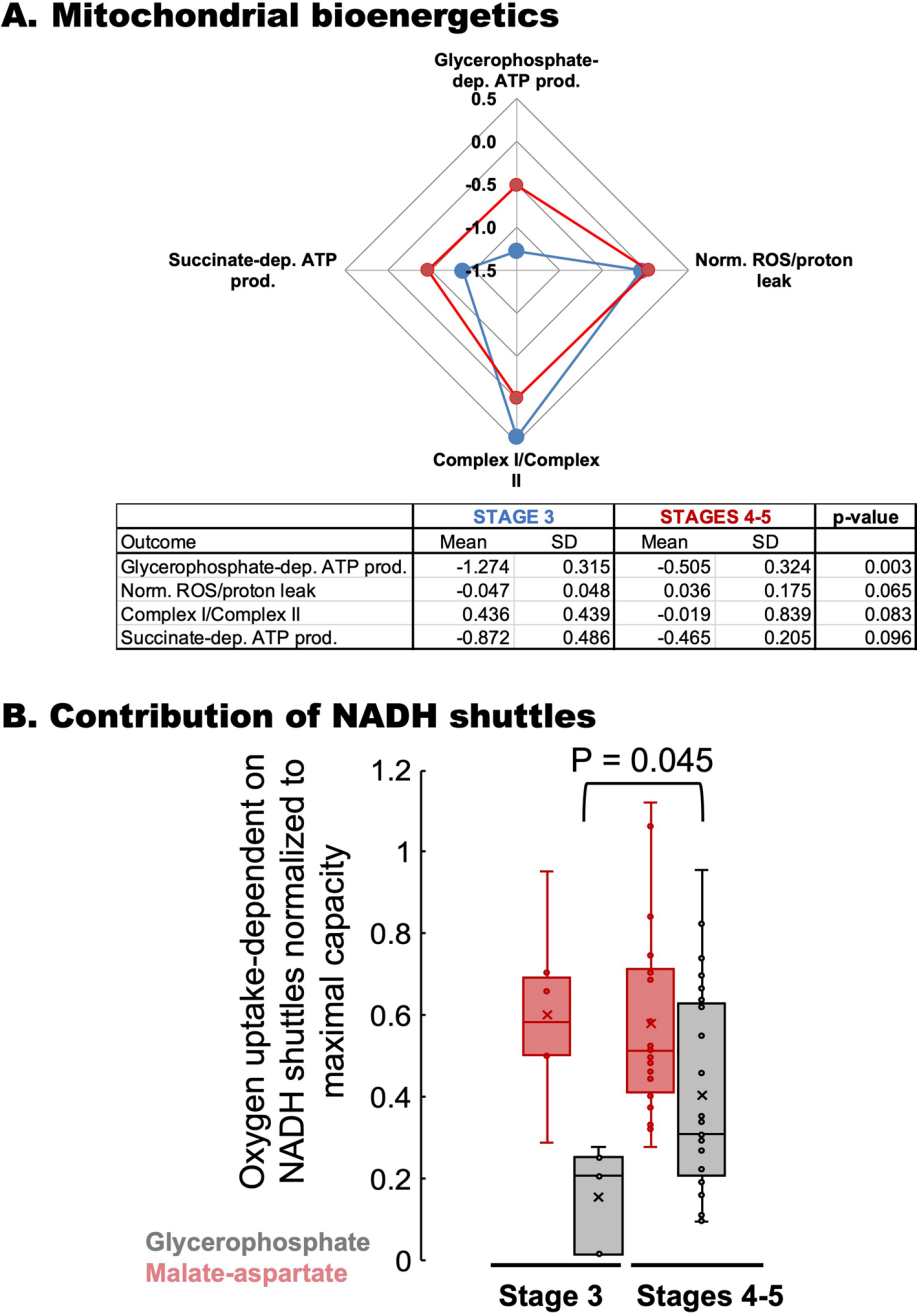
Bioenergetics of PBMCs from patients at Stages 3 vs. 4–5. (**A**) Mitochondrial bioenergetic outcomes from PBMCs obtained from carriers and assigned by the VRE algorithm at Stage 3 (Fig. 2C, box C) and Stages 4–5 (Fig. 2C, box D) were plotted using a spiderweb chart. For simplicity, only those with *p* ≤ 0.1 were shown. Bottom panel: Statistical analyses performed by using an unpaired two-tailed Student’s *t* test with unequal variances. *dep.* Dependent, *prod.* Production, *Norm.* normalized to glucose–glutamine respiration. (**B**) Contribution of NADH shuttles: The oxygen-linked ATP production fueled by glucose–glutamine of primary fibroblasts from carriers (Malate-aspartate in red) was taken as a surrogate for the contribution of the malate-aspartate shuttle. The oxygen-linked ATP production fueled by glycerol-3-phosphate (GP in grey) was taken as the contribution of the glycerophosphate shuttle. Both rates were normalized to the maximum respiratory rate^120^. A two-tailed Student *t* test for unpaired data with unequal variance was run for GP with no statistical difference [*t*(29) = 0.203; *p* = 0.840] but with statistical significance for Malate-Aspartate [*t*(26) = − 2.102; *p* = 0.045]. Data were visualized by using a box plot. Each box enclosed 50% of the data (closed or open circles) with the median value of the variable displayed as a line and the mean as an x. The top and bottom of the box mark the limits of ± 25% of the variable population. The lines extending from the top and bottom of each box mark the minimum and maximum values within the dataset that fall within an acceptable range. Any value outside this range, called an outlier, is displayed as an individual point whose value is outside the upper quartile + 1.5 × interquartile range and lower quartile − 1.5 × interquartile range. The height of each box represents the interquartile range.

Mitochondrial bioenergetic differences were observed between carriers at Stages 0–1 vs. Stages 2–3 (Fig. 2C, boxes labeled as A and B) and between those at Stage 3 vs. Stages 4–5 (Fig. 2C, boxes labeled as C and D). In this regard, a higher glycerophosphate-fueled ATP production was observed in PBMCs from carriers at Stages 4–5 than those at Stage 3. Confirming the decision tree classification, those PBMCs from carriers at higher stages showed higher glycerol-3-phosphate-fueled ATP production (to a lesser extent with succinate) than those at Stage 3 (Fig. 5A). Similar to bioenergetic data from patients from Stages 0–1 vs. those at Stages 2–3, differences between Stages 3 vs. 4–5 included a higher mtROS production and altered Complex ratios, but with no changes in either Complex IV or citrate synthase activities.

The glycerol-3-phosphate-fueled ATP production is mediated by the coordinated action of the mitochondrial glycerol-3-phosphate dehydrogenase (mGPDH) with a distinct, cytosolic cGPDH to transfer reducing equivalents from NADH—generated at the glycolytic step catalyzed by glyceraldehyde dehydrogenase—into the mitochondrial respiratory chain through the glycerophosphate shuttle. The reoxidation of NADH, critical for sustaining glycolysis, is achieved not only by this shuttle but also by the malate-aspartate shuttle and lactate dehydrogenase^87^. To estimate the contribution of the shuttles glycerophosphate and malate-aspartate (both contributing to transferring reducing equivalents to the mitochondrial electron transport chain), the oxygen-linked ATP production rates sustained by glycerol-3-phosphate or basal (glucose–glutamine) were normalized to the maximum respiratory rate (Fig. 5B). While the activity of the malate-aspartate shuttle was similar between Stages 3 and 4–5 when PBMCs utilize glucose–glutamine, that of the glycerol-3-phosphate one was higher in those at higher stages. Taken together, at higher stages, there is higher lipolysis (with an increased flux of glycerol) at the expense of higher mtROS as mGPDH participates significantly in the mitochondrial ROS production^87–89^.

**Figure 6:**
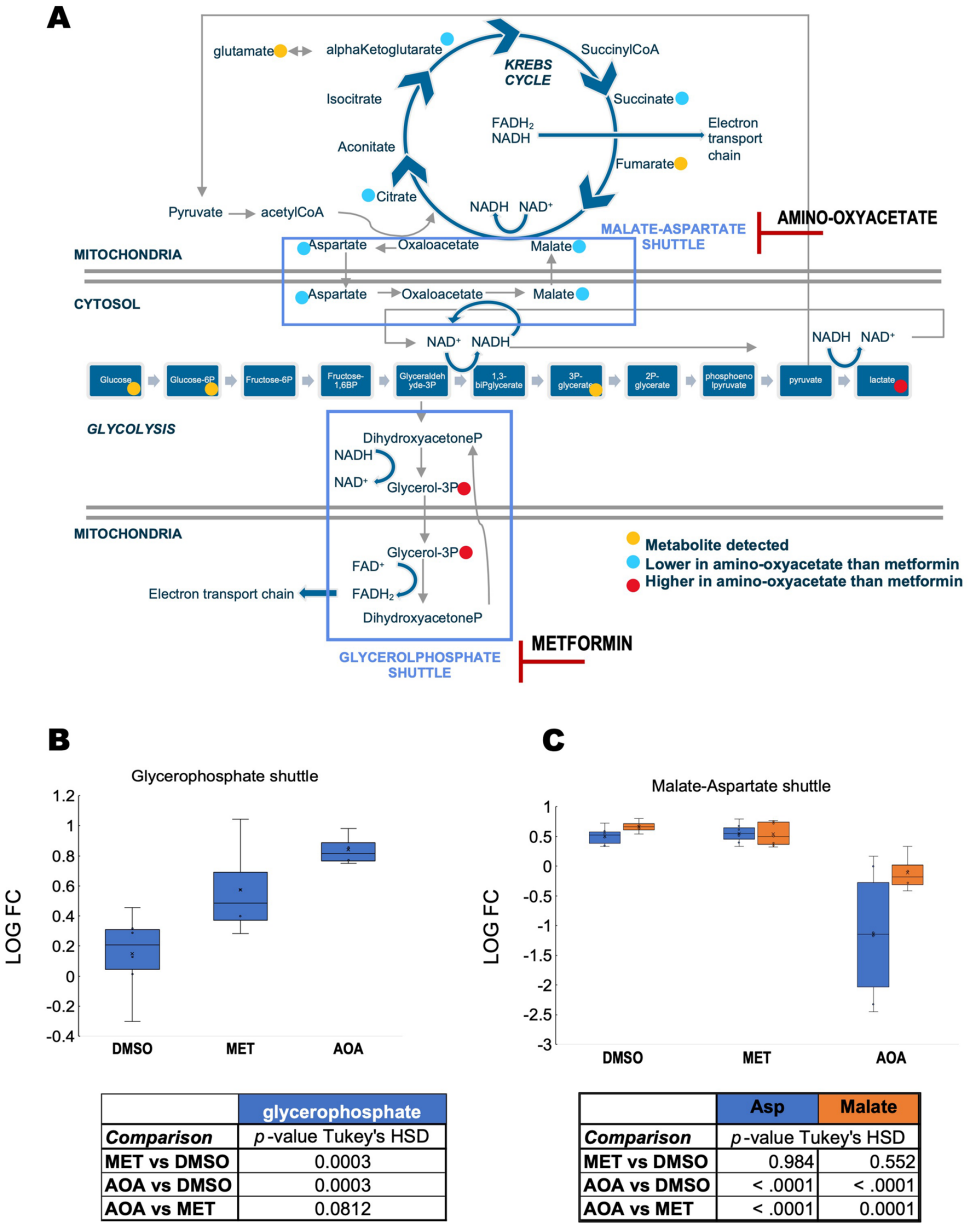
Metformin-dependent inhibition of glycerophosphate shuttle and amino-oxyacetate inhibits the malate-aspartate shuttle in fibroblasts from FXTAS carriers with high mitochondrial glycerol-3-phosphate dehydrogenase activity. Four primary fibroblasts (50 µM metformin or MET, 5 mM amino-oxyacetate or AOA) were treated for 24 h. Their metabolome and mitochondrial bioenergetic capacity were compared to DMSO-treated as indicated in the “Methods”. Panel (**A**): Inhibition sites for metformin and amino-oxyacetate. The inhibition exerted by these compounds is shown in the context of glycolysis, Krebs’ cycle, and malate-aspartate and glycerophosphate shuttles. Panel (**B**): Levels of glycerol-3-phosphate were evaluated by metabolomics under each condition (vehicle or dimethylsulfoxide, DMSO; *MET* metformin; *AOA* amino-oxyacetate) as a proxy for glycerophosphate shuttle. Panel (**C**): Malate-aspartate shuttle components (Aspartate or Asp in blue; malate in orange). For panels (**B**,**C**): Data were visualized by using a box plot (box height is the interquartile range; top and bottom of boxes are the upper and lower quartiles; top and bottom lines represent the maximum and minimum values; median is shown as a line; average as an x). Tables at the bottom of each panel show the *p*-values obtained from Tukey’s honestly significant difference (HSD) for each comparison and show only outcomes with *p*-values ≤ 0.1. Matched-samples ANOVA was run to test for statistical significance across treatments followed; if *p* ≤ 0.05, a Tukey’s HSD post-test was run. All analyses were performed with SPSS.

**Figure 7:**
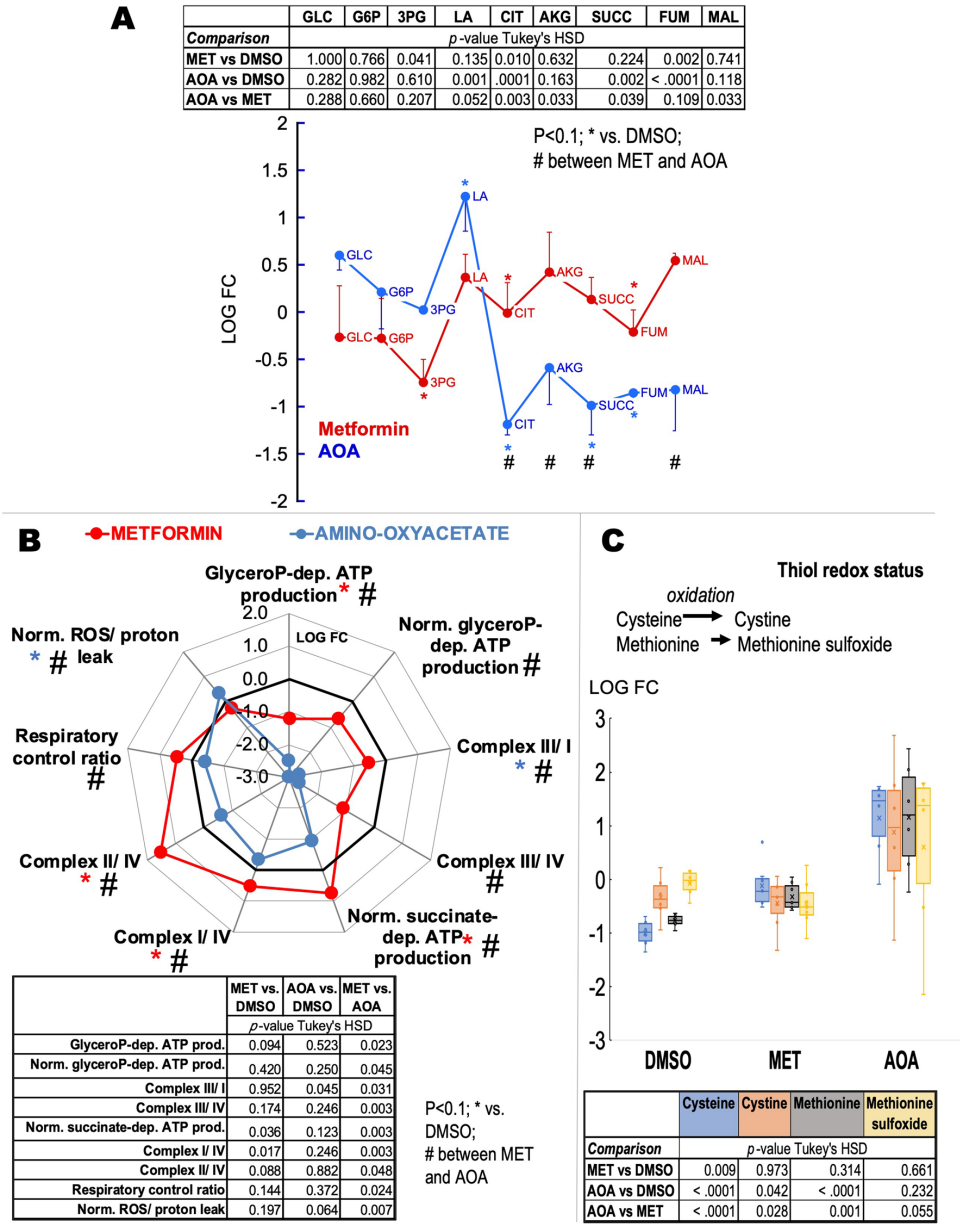
Metformin-dependent inhibition of glycerophosphate shuttle improves bioenergetic outcomes and decreases oxidative stress of fibroblasts from FXTAS carriers with high mitochondrial glycerol-3-phosphate dehydrogenase activity. Panel (**A**): Glucose catabolism, including glycolysis and Krebs’ cycle, was evaluated by assessing key metabolites by using metabolomics. The metabolites were obtained from four primary fibroblasts (50 µM metformin or MET, 5 mM amino-oxyacetate or AOA) treated for 24 h. For simplicity, only one-side error bars are shown, representing the standard error of the mean. P-values obtained using Tukey’s HSD are shown with an asterisk (if the comparison was against DMSO) or # (if the comparison was between MET and AOA). *GLC* glucose, *G6P* glucose-6-phosphate, *3PG* 3-phosphoglycerate, *LA* lactic acid, *CIT* citrate, *AKG* alpha-ketoglutarate, *SUCC* succinate, *FUM* fumarate, *MAL* malate. Panel (**B**): Bioenergetic outcomes-Spider plot showing bioenergetic outcomes of fibroblasts from four carriers treated with metformin or amino-oxyacetate as indicated above. Metformin data are shown in red; amino-oxyacetate in blue. *glyceroP* glycerophosphate, *dep.* dependent, *prod.* Production, *Norm.* normalized by the activity of citrate synthase except ROS/proton leak normalized to oxygen-linked ATP production. P-values obtained using Tukey’s HSD are shown with an asterisk (if the comparison was against DMSO) or # (if the comparison was between MET and AOA). Panel (**C**): Thiol redox status was assessed by the levels of sulfur-containing amino acids, which were determined by mass spectrometry as described before^28,29^. Data were visualized by using a box plot (box height is the interquartile range; top and bottom of boxes are the upper and lower quartiles; top and bottom lines represent the maximum and minimum values; the median is shown as a line; average as an x). For all panels: Tables on each panel show the *p*-values obtained from Tukey’s honestly significant difference (HSD) for each comparison; shown only outcomes with *p*-values ≤ 0.1. Matched-samples ANOVA was run to test for statistical significance across treatments followed; if *p* ≤ 0.05, a Tukey’s HSD post-test was run. All analyses were performed with SPSS.

To test whether the bioenergetics of carriers with higher glycerol-3-phosphate-fueled ATP production would benefit from lowering the activity of the glycerol-3-phosphate shuttle, we treated fibroblasts from four carriers (3 males, one female, all within 67–68 years of age) selected from the decision tree classification (Fig. 2C labeled D as they had relatively higher glycerol-3-phosphate activity) with either metformin (50 µM) or amino-oxyacetate. The rationale of this strategy was that bioenergetic improvements would be obtained upon inhibiting directly (metformin-dependent inhibition of mGDPH^90^) or indirectly (inhibitor of the malate-aspartate shuttle^91,92^ that disrupts the NADH/NAD^+^ ratio) the glycerophosphate shuttle (Fig. 6A). In this experimental setting, we used biologically relevant metformin concentrations within the 10–40 μM plasma levels from patients treated with 1 g metformin twice a day^93^ and within the 74–100 µM detected in plasma and livers from rats treated acutely with 50 mg/kg body weight^90^. While it is known that metformin may exhibit a plethora of effects^94,95^, at these relatively low concentrations, and similar to other studies, no changes in Complex I activity were observed, and the incubation period did not allow for following putative changes in mitochondrial biogenesis (assessed by citrate synthase activity). As expected, both treatments increased levels of glycerol-3-phosphate compared to vehicle-treated ones (Fig. 6B), while only amino-oxyacetate disrupted metabolites within the malate-aspartate shuttle (Fig. 6C). Both inhibitors showed similar patterns of glucose metabolism, i.e., higher lactic acid and decreases in Krebs’ cycle intermediates, with more profound and significant changes observed for amino-oxyacetate than metformin (Fig. 7A). The lower levels of Krebs’ cycle intermediates may result from disrupted anaplerotic reactions (i.e., reactions that serve to replenish Krebs’ cycle intermediates) affected by the imbalances in cytosolic NADH/NAD^+^ status. These results indicated that by inhibiting either shuttle (but quantitatively more the malate-aspartate one), the reduced equivalents produced during glycolysis are kept in the cytosol in the form of lactic acid via the activity of lactate dehydrogenase.

As predicted, the mitochondrial bioenergetic analysis indicated that both treatments (albeit metformin at higher degrees) decreased the ATP production sustained by glycerophosphate (normalized or not to citrate synthase); as such, those Complex ratios in which Complex III was either a denominator or numerator changed accordingly. Changes specific to metformin were the increase in the succinate-dependent ATP production, Complex I/IV and II/IV ratios, respiratory control ratio, and a decrease in ROS/proton leak production. Taken together, metformin vs. amino-oxyacetate treatment was beneficial in those fibroblasts with relatively high glycerol-3-phosphate-fueled ATP production because it decreased the rate of ROS, increased the rate of ATP production fueled by succinate, and increased the coupling between electron transfer and ATP production. Conversely, amino-oxyacetate vs. vehicle treatment increased the rate of ROS production normalized to basal respiration and dramatically decreased the Complex III/Complex I ratio (Fig. 7B). As such, differences between metformin and amino-oxyacetate treatments were essentially based on proton leak and mtROS production and the segment of the electron transport chain between the entry points of succinate and glycerophosphate.

To test the impact of oxidative stress by inhibiting either the malate-aspartate or the glycerol-3-phosphate shuttle, we evaluated the levels of cysteine, methionine, and their oxidized products (cystine and methionine sulfoxide, respectively) as sulfur-containing amino acids are strongly affected by oxidative stress (Fig. 7C). Metformin significantly increased cysteine levels with no changes in methionine or their respective oxidized products, suggesting that by decreasing mGPDH-mediated ROS production, reduced cysteine levels were spared. These results are consistent with the lower mtROS production indicated above achieved by metformin treatment. As expected for a condition resembling nonphosphorylating mitochondria due to the shortage of NADH supply via the malate-aspartate shuttle, amino-oxyacetate treatment increased the mtROS production compared to vehicle-treated cells. However, despite the increase in oxidative stress, the levels of cysteine and methionine were higher than those with the vehicle (Fig. 7C). This can be understood by the aminooxyacetate-dependent inhibition of cystathionine gamma-lyase^96^, a key enzyme in the catabolism of these amino acids.

## Discussion

In this study, we tested whether ANN approaches could aid in diagnosing FXTAS stages based on age, sex, MRI findings, and bioenergetic outcomes from two cell types, PBMC and fibroblasts. Our results indicated that the 3 ANN approaches utilized here were able to make a suitable stage classification with an accuracy of 86.27% (SFAM), 76% (backpropagation), and 41.67% (VRE) based only on two MRI and four bioenergetic findings—both experimental, unbiased outcomes—of the many outcomes included in the study, all of which were objective measures of brain imaging and cellular bioenergetic assessments.

Although the progression of FXTAS increases in older male carriers, age was not among the critical factors expected for predicting the progression of FXTAS because, in our study, we stratified the sampling for the training and test sets, in which age and sex were proportionally represented. Notably, although sex was also not among the main discriminators for predicting FXTAS stages as expected for an X-linked disorder, on average, the ratio of male-to-female mismatch ratio was 1.45 across the three methods tested here, supporting the notion that women may be protected by the second X chromosome^97^ and estrogen^98,99^.

The decision tree classification obtained by using VRE algorithm utilized two of the five brain imaging outcomes, i.e., WMHs and whole brain volumes normalized by intracranial volume, for predicting FXTAS stages. As indicated before, white matter lesions presented as WMHs on T2 and FLAIR images in the middle cerebellar peduncles, brainstem, deep white matter, and corpus callosum are important radiologic biomarkers for FXTAS diagnosis. Neuropathological studies revealed that white matter disease is characterized by marked spongiosis, loss of axons, and demyelination in the cerebrum and cerebellum in most brains of carriers with FXTAS^11,12^. However, WMHs are not unique to FXTAS as they have also been observed in aging and other neurodegenerative diseases^100–103^. Conversely, bilateral WMHs in the middle cerebellar peduncles are much rarer and present in FXTAS-affected patients but also in some patients with Leigh syndrome, brain stroke, demyelination, multiple system atrophy-cerebellar variants, spinocerebellar ataxia, and leukoencephalopathy^51,104–106^.

In the decision tree, the values of WMHs normalized by intracranial volume helped classify FXTAS Stages 0–3 (≤ 12,600 mm^3^) versus 3–5 (> 12,600 mm^3^). In contrast, the classification of stages 3 versus 1, 2, and 4 relied on both imaging (normalized whole brain volume) and bioenergetic outcomes (Fig. 2C). This result is consistent with our recent publication reporting a significant correlation between WMH volume and FXTAS stage in 74 premutation carriers (54% males; aged ≥ 50 years) after adjusting for age, sex, and intracranial volume, although substantial overlapping in WMHs volume across all FXTAS stages can be observed^15^. Values of WMHs normalized by intracranial volume < 600 mm^3^ help classify carriers at Stages 0–1, whereas those with values between 600 mm^3^ and 12,600 mm^3^ and normalized whole brain volume ≤ 1.3 dm^3^ predicted a Stage 2 diagnosis. Bioenergetic outcomes were necessary for distinguishing among Stages 0–1, 2, and 3 for those with normalized whole brain volume > 1.3 dm^3^. This is also consistent with our previous finding of age-related accelerated brain volume loss in carriers without FXTAS than noncarriers, which may become more evident as FXTAS develops^16^.

Intriguingly, the two binary variables, the presence of WMHs in the middle cerebellar peduncles and the cerebellum-brainstem, were not selected by ANN for FXTAS stage classification. In our recent study of 175 carriers and 82 controls aged ≥ 45 years^54^, the WMHs presence in the middle cerebellar peduncles was detected only in the premutation carriers (52%) with significant correlations with impaired motor and executive function in those carriers with > 75 CGG repeats. In the current study of 111 carriers, the prevalence of WMHs in the middle cerebellar peduncles and WMHs in the cerebellum and brainstem increased with FXTAS stage (middle cerebellar peduncles/cerebellum and brainstem: Stage 0–1, 6.1%/57.6%; Stage 2, 25%/80%; Stage 3, 42.1%/94.7; Stages 4–5, 80%/100%). It is conceivable that WMH volume, providing a fine-grained measurement for white matter damage, is more useful for stage classification than the information provided by two binary variables. Similarly, ventricular volume was not selected by ANN as it may carry overlapping information with the WMH volume, further evidenced by their significant positive correlation [Pearson’s *r* (109) = 0.598, *p* < 0.0001].

**Figure 8:**
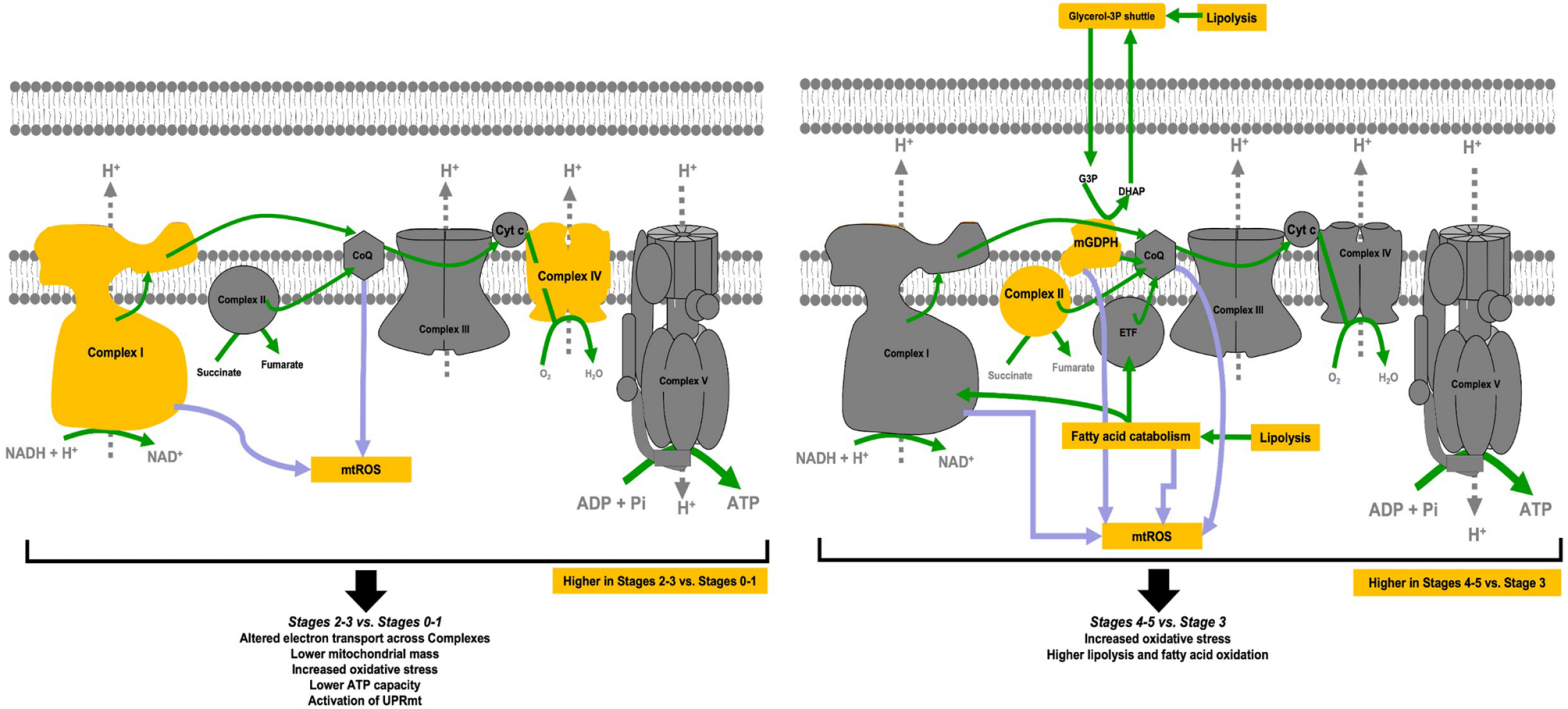
Bioenergetic differences across stages. Changes in bioenergetics (in yellow) that match the assignment of stages according to the decision tree classification (Fig. 2C). Altered Complex ratio and increased mitochondrial ROS characterized Stages 2–3 vs. Stages 0–1 (left panel), accompanied by lower mitochondrial mass (citrate synthase activity; see text). These changes result in an overall lower ATP production (altered Complex ratio and lower mitochondrial mass), and may activate the mitochondrial unfolded protein response (UPRmt). Higher fatty acid oxidation (Complex II, mGDPH) and mitochondrial ROS characterized Stages 4–5 vs. Stage 3. While these changes may increase ATP derived from mitochondrial fatty acid beta-oxidation, they may increase the susceptibility of neurons to hypoxic events and to ATP deficits as glucose catabolism is faster than that from fatty acids. Arrows in green indicate the electron flow and direction of biological processes; purple arrows indicate the ROS sources. *CoQ* coenzyme Q, *Cyt c* cytochrome *c*, *Pi* phosphate, *mGDPH* mitochondrial glycerophosphate dehydrogenase, *ETF* electron transfer flavoprotein, *G3P* glycerol-3-phosphate, *DHAP* dihydroxyacetone-phosphate.

Regarding bioenergetic outcomes selected by ANN to discriminate between early and late stages, it is remarkable that one of the outcomes (Complex IV) was affected in both cell types (bioenergetic outcomes summarized in Fig. 8). The consistency in the outcome Complex IV activity between fibroblasts and PBMCs indicates a conserved mechanism despite the apparent differences between the cell types, namely, proliferative vs. quiescent, grown under optimal cell culture conditions vs. reflecting the carrier’s homeostasis within days to several weeks before blood extraction, germ layer of origin ectoderm vs. endoderm. The results indicate that cells from carriers at Stages 0–3 may have a mitochondrial-nuclear imbalance evidenced by changes in Complex IV activity because this Complex is encoded by the coordinated action of both the nuclear and the mitochondrial genomes. As indicated before, an altered ratio of mitochondrial Complexes ensues in not only deficits in ATP production but also in the activation of UPRmt^85^. This is supported by the activation of UPRmt in worms and mammals in response to the stoichiometric imbalance between nuclear DNA- and mitochondrial DNA-encoded oxidative phosphorylation proteins, also known as mitochondrial-nuclear protein imbalance^85^. This scenario extends our previous reports on a disrupted proteostasis in the *FMR1* premutation^24,39,107^ with an accumulation of oxidatively modified proteins and deficits in mitochondrial protein import, processing, and folding^21–23^. Notably, the apparent functional connection between mitochondrial-nuclear protein imbalance and UPRmt, and their link to aging^85^ may prompt future research to explore whether targeting UPRmt can prevent or slow down age-dependent neurodegeneration.

While increases in mtROS (oxidative stress) seem to underlie all transitions from early to late stages, the increases in glycerol-3-phosphate shuttle in Stages 4–5 may represent an attempt to overcome energy failure by increasing glycerol and fatty acid oxidation at the expense of increased oxidative stress (Fig. 8). This is a crucial point as it indicates that controlling mGPDH and oxidative stress (especially cumulative damage over time) may be critical to preventing the transition of FXTAS carriers to higher stages. As indicated above, while the upregulation of glycerophosphate-mediated ATP production in cells from carriers at higher FXTAS stages may be interpreted as an attempt to supply ATP via mitochondrial fatty acid beta-oxidation, several factors need to be considered to infer that this attempt might be detrimental to carriers at higher stages. Among them were (i) the inherent higher mtROS production of both mGPDH activity and fatty acid beta-oxidation, (ii) the higher glycolytic capacity of cells with high mGPDH expression^108^, which may limit the indispensable mitochondria-derived ATP production in neurons, and (iii) the slower ATP production and higher oxygen demand to oxidize fatty acids vs. glucose which may not be matched by the high demand of rapid and sustained neuronal firing, putting neurons at risk of hypoxic events^109^. In support of these concepts, in this study, metformin-dependent inhibition of mGPDH decreased mtROS production, improved the coupling and increased the ATP production sustained by succinate (Fig. 7B). Treatment of fibroblasts from carriers at Stages 3–4 with the potent NRF2 activator sulforaphane successfully recovered all bioenergetic outcomes highlighting the need for enhancing UPR and antioxidant responses to protect mitochondria from further damage^39^.

Limitations of this study include the lack of testing for the specificity of these ANN analyses in other diseases displaying a psychiatric and cognitive performance pattern overlapping with FXTAS (e.g., several spinocerebellar ataxias and frontotemporal dementia). Due to sample availability and cell number, some bioenergetic data were missing from a few samples and were estimated with suitable algorithms. All participants were recruited from one facility (MIND Institute), leading to potential sample bias. Finally, most male carriers included in this study were at Stage 3, which may limit the applicability to other stages.

Despite the aforementioned limitations, this study delineates a strong molecular signature in detecting FXTAS across stages, offering the best therapeutic intervention window. If validated in prospective larger cohorts, the predicted trajectories through the implementation of a neural network will represent a breakthrough in clinical screening and swift detection of FXTAS morbidity in carriers of the *FMR1* premutation without overt FXTAS symptoms or to prevent the transition to stages with more detrimental symptoms and co-morbidities.

## Methods

### Study design and participants

Written informed consent was obtained from all participants before participation in line with the Declaration of Helsinki. The study was reviewed and approved by the Institutional Review Board of the University of California Davis Medical Center, and all methods were performed by their guidelines and regulations.

The present study consisted of a retrospective chart review of adult patients with FXTAS. Participants were carriers with the premutation enrolled in a large research study at the UC Davis Medical Investigation of Neurodevelopmental Disorders (MIND) Institute. We included individuals who participated in the Genotype–Phenotype Study of Families with Fragile X (NICHD HD036071) from 2013 through 2019 and with the availabilities of MRI, primary skin fibroblasts, and blood samples. The inclusion criteria required that participants were noncarriers (n = 9) or carriers of the premutation (n = 89), all adults (≥ 18 years), and both sexes included. Among the carriers, they would have a definite, probable, or possible FXTAS diagnosis in clinical Stages 1–5. We considered FXTAS Stage 0 for a carrier with no FXTAS diagnosis. FXTAS Stage^7^ was scored by a trained physician (R.J.H.) based on the severity of movement and gait impairments (1: subtle or questionable signs, 2–6: evident tremor/balance problems with minor to severe interference of daily living). Only those with the most outcomes were considered if multiple visits were available. Only the oldest was included if two or more visits had the most outcomes. As such, no longitudinal data were taken into consideration. Exclusion criteria were other neurodegenerative dementias, a history of cerebrovascular accidents, traumatic brain injury, and alcohol abuse or dependence. A total of 127 participants (both genders) were included in the final data analysis, and their demographics are summarized in Fig. 1.

### Chemicals and biochemicals

EDTA, EGTA, KH_2_PO_4,_ sodium succinate, digitonin, rotenone, antimycin A, oligomycin, malonate, ascorbic acid, *N,N,N*′*,N*′*-*tetramethyl-*p-*phenylenediamine, KCN, HEPES, metformin and AOA were purchased from Sigma (St Louis, MO, USA). Tris–HCl, glycine, sodium chloride, and KCl were purchased from Fisher (Pittsburg, PA, USA). Bovine serum albumin (fatty acid-free) was obtained from MP Biomedicals. All reagents were of analytical grade or higher.

### PBMCs preparation

PBMCs were isolated from blood samples (5–7 ml) collected in BD Vacutainer™ glass mononuclear cell preparation tubes (BD Biosciences, Franklin Lakes, NJ, USA) as previously described^25^. Lymphocyte suspensions were pelleted by centrifugation for 1 min at 2000 rpm in a microfuge at 4 °C. The supernatants were discarded, and cell pellets were used immediately to evaluate mitochondrial outcomes.

### Culture of primary skin fibroblasts

Skin biopsies were obtained from participants from the same skin area (punch biopsy on left upper back) to minimize variances due to body site or different exposure to the external milieu. To minimize confounding factors, such as significant telomere shortening across passages and corresponding features of replicative senescence, we used fibroblasts at their earliest passage available (on average 4–5). This approach also prevented the putative instability of cultured cells from subjects with respiratory chain disorders, which tend to return to normal values across passages because cells less affected may proliferate faster than those more severely affected^110^. Fibroblasts were obtained from Dr. P. Hagerman’s laboratory with the help of Ms. Glenda Jackson. Fibroblasts were grown in high glucose Minimum Essential Medium supplemented with 15% fetal bovine serum, 2 mM glutamine, and 1 mM sodium pyruvate, as previously described^22^.

Metformin and amino-oxyacetate treatments were performed on primary fibroblasts from four carriers with FXTAS, three males (2 at Stage 3 and one at Stage 4) and one female (Stage 3) with a median (range) age of 67 (67–68). This subset was selected randomly from patients within box D as they presented relatively higher activity of the glycerol-3-phosphate shuttle (Fig. 2C). For the treatment with metformin and AOA, cells were grown at ~ 50% confluence and treated with 50 µM metformin, 5 mM AOA, or with the equivalent volume of DMSO for 24 h, detached by trypsinization (ThermoFisher Scientific), and assessed for viability by trypan blue with a TC20 cell counter (BioRad). Bioenergetic outcomes were determined as it is detailed below.

### Mitochondrial outcomes of PBMCs and fibroblasts

Mitochondria-dependent oxygen consumption was evaluated in either fibroblasts^27^ or freshly isolated lymphocytes permeabilized with digitonin as previously described^36^. Briefly, lymphocytes (0.5–1.0 × 10^6^) or fibroblasts (2.0 × 10^6^) were added to the Clark-type oxygen electrode chamber containing 0.3 ml of a buffer (0.22 M sucrose, 50 mM KCl, 1 mM EDTA, 10 mM KH_2_PO_4_, and 10 mM HEPES, pH 7.4). After establishing the baseline, the oxygen consumption rates were evaluated with (i) 1 mM ADP, 1 mM malate-10 mM glutamate for 5–8 min until reaching a steady rate, followed by the addition of 5 μM rotenone to inhibit Complex I; (ii) 10 mM succinate for 5–8 min until reaching a steady rate, followed by adding 1 mM malonate and 3.6 μM antimycin A; and (iii) 10 mM ascorbate and 0.2 mM *N,N,N*′*,N*′-tetramethyl-*p*-phenylenediamine followed by the addition of 1 mM KCN to inhibit Complex IV. The succinate-fueled oxygen uptake and cytochrome *c* oxidase activity were evaluated as the difference in oxygen consumption rate recorded before and after the addition of the respective inhibitors, malonate, and KCN. The respiratory control ratio (RCRu) was calculated as the quotient of oxygen consumption rates of intact cells in 10 mM glucose (present in RPMI-1640) in State 3u (with 2 µM carbonylcyanide-*p*-trifluoromethoxyphenylhydrazone; FCCP) and that of State 4 (with 0.2 µM oligomycin). SRC (spare respiratory capacity) was calculated as the ratio (in percentage) between State 3u and basal respiration.

In contrast, ROS/proton leak was calculated as the ratio (in percentage) between State 4 and basal respiration. Protein concentration was determined by the Lowry method and calculated according to the bovine serum albumin standard curve. Citrate synthase activity was evaluated at 412 nm in both cell types with a Tecan Infinite M200 microplate reader equipped with the Magellan software (Austria)^36^. All carrier parameters were normalized to the pooled noncarrier values. For some samples (< 5), bioenergetic data from either fibroblasts or PBMC were missing (sample loss, not enough cells for reliable detection, among others). In those cases, missing data were estimated using the feature *k*-nearest-neighbor imputation^111^.

### MRI acquisition and processing

All methods had been described in detail before^15^. MRI scans were acquired on a Siemens Trio 3 T MRI scanner equipped with a 32-channel head coil (Siemens Medical Solutions, Erlangen, Germany). One-millimeter isotropic T1-weighted scans were collected covering the whole brain using the magnetization-prepared rapid gradient-echo sequence in 192 sagittal slices with a repetition time of 2170 ms, echo time of 4.82 ms, and 7° flip angle. Fluid attenuated inversion recovery (FLAIR) images for quantifying WMHs were acquired in 104 sagittal slices of 1.9-mm thickness with an in-plane resolution of 0.47 mm^2^, repetition time of 5000 ms, echo time of 456 ms, and inversion time 1700 ms. Both T1 and FLAIR scans were corrected for intensity inhomogeneities due to the MRI bias field using N4^112^. Whole-brain volume, ventricular volume, and brain scaling factor (for correcting individual differences in cranial size) were obtained on magnetization-prepared rapid gradient-echo scans using the SIENAX program^113^ from FSL. Optimal values were obtained by adjusting the parameter used by the BET program for brain extraction. Whole-brain WMH volume was quantified on FLAIR images using a lesion prediction algorithm from SPM12^114^. Lesion masks were generated by setting appropriate thresholds on lesion probability maps using the FSL command, fslmaths, followed by manual correction for errors using ITK-Snap^115^. Volume calculation of WMH was performed using the FSL command, fslstats. Normalized volumes of the whole brain, ventricles, and WMHs normalized by intracranial volume were computed by their multiplication with the brain scaling factor. Binary variables were coded according to the presence or absence of WMHs in the middle cerebellar peduncles and cerebellar whiter matter, respectively, based on the generated WMHs lesion mask. Cerebellar white matter included the middle cerebellar peduncles, transverse pontine fiber in the pons, superior cerebellar peduncle, and its decussation in the midbrain.

### Artificial neural network (ANN) for FXTAS stage diagnosis

The ANN approach applied to this study followed that described before for Wilson’s disease^50^. The ANN design consisted of a three-layer network: an input layer with either 2 or 5 units for defining FXTAS Stages (Fig. 1A), sex, age, five units of brain imaging (as indicated above), and 22 units of mitochondrial outcomes for each cell type (PBMC and fibroblasts); a hidden layer, with four units; and an output layer for the FXTAS stage. The simplified fuzzy adaptive resonance theory map (SFAM) algorithm was used for the prediction of “early” (noncarriers and carriers at Stages 0–2) and “late” (carriers at Stage ≥ 3), whereas the backpropagation algorithm was used to predict numerical values of FXTAS stages (Fig. 1A) (NeuNetPro software; CorMac Technologies Inc., Canada). The unsupervised training variables included all of those indicated above. To select the training set size for each analysis, we performed a learning curve analysis^49^. We withdrew a relatively small random sample from our data to train an ANN and used this training set to predict a randomly sampled test set. Then, the size of our training sample was increased while keeping the same test set to track the degree to which predictive accuracy on the fixed test set increased with the training set size. This iterative process resulted in a training data size that minimized the differences between the network classifications and the clinical diagnoses. We repeated the learning curve analysis multiple times with different test set samples to reduce variation in predictive accuracy (85 samples were used for training, and 42 were used for testing with SFAM and backprop). The last algorithm (Visual Rule Extraction or VRE)^81,82^ utilized the patterns of input factors associated with stages. It was trained with a randomly selected sample of 75 carriers from the 111 premutation carriers, all with known clinical status. This set of 75 carriers was selected using stratified random sampling (Python package; SciKitLearn) to ensure that each subgroup within the population received proper representation within the validation sample. The stratified random sampling provides better population coverage since it allows control over the subgroups to ensure that all of them are represented in the sample. Once the network was trained, the remaining 36 carriers were "tested" using the trained network. The neural network classifications were then compared with the known clinical diagnoses to determine whether the network could verify disease status reliably. The output was visualized with a decision tree with adjustable tree pruning (55% pruning with a minimum of five participants).

### Statistical analysis

The authors were blinded to the diagnosis of the subjects until all data were collected. If data for a subject were available from multiple visits, the oldest or the one with the most outcomes was taken. As such, no longitudinal data were taken into consideration for this study. Bioenergetic outcomes in fibroblasts and PBMCs were evaluated in at least triplicates. The statistical analyses involved comparisons between boxes or before and after treatments. The treatments (DMSO, metformin, and AOA) were compared using matched-samples 1-way ANOVA with Tukey’s multiple comparisons post-test. Differences between boxes were analyzed via an unpaired, two-tailed Student’s *t* test with unequal variances. The variance equality was confirmed by using Levene’s test^116–118^. For every comparison, a power analysis was run in which the alpha and power of the experiment were imputed, given the beta/alpha ratio, sample size, and effect size. For a two-tailed *t* test (difference between two independent means), an effect size of 2, beta/alpha ratio = 1, the computed alpha values ranged from 0.087 to 0.176 with a power of 0.823 to 0.913, highlighting that the *p*-values of 0.1 are within the alpha range. Fisher exact test and Pearson correlation were performed with SPSS. For boxes’ analyses, points whose values were either greater than upper quartile 2 × interquartile distance or less than lower quartile − 2 × interquartile distance were considered outliers and excluded from further analysis. The data statistical software SPSS 27.0.0 and the data analysis software GraphPad Prism 9 were used to analyze the experimental data.

### Institutional review board statement

The University of California Davis Institutional Review Board and the State of California Committee for the Protection of Human Subjects approved this study.

### Informed consent

Neither data nor specimens were collected until written informed consent was obtained from the patients. Written informed consent has been obtained from the patient(s) to publish this paper.

## Data Availability

The datasets used and analyzed during the current study are available from the corresponding author upon reasonable request.
